# Dynamic social interactions and keystone species shape the diversity and stability of mixed-species biofilms – an example from dairy isolates

**DOI:** 10.1038/s43705-023-00328-3

**Published:** 2023-11-15

**Authors:** Faizan Ahmed Sadiq, Koen De Reu, Hans Steenackers, Ann Van de Walle, Mette Burmølle, Marc Heyndrickx

**Affiliations:** 1 Flanders Research Institute for Agriculture, Fisheries and Food (ILVO), Technology and Food Science Unit, Brusselsesteenweg 370, 9090 Melle, Belgium; 2 Centre of Microbial and Plant Genetics (CMPG), Department of Microbial and Molecular Systems, KU Leuven, Kasteelpark Arenberg 20, 3001 Leuven, Belgium; 3 Section of Microbiology, Department of Biology, University of Copenhagen, Universitetsparken 15, 2100 Copenhagen, Denmark; 4 Ghent University, Department of Pathobiology, Pharmacology and Zoological Medicine, Salisburylaan 133, B-9820 Merelbeke, Belgium

**Keywords:** Biofilms, Environmental microbiology, Microbial ecology

## Abstract

Identifying interspecies interactions in mixed-species biofilms is a key challenge in microbial ecology and is of paramount importance given that interactions govern community functionality and stability. We previously reported a bacterial four-species biofilm model comprising *Stenotrophomonas rhizophila*, *Bacillus licheniformis*, *Microbacterium lacticum*, and *Calidifontibacter indicus* that were isolated from the surface of a dairy pasteuriser after cleaning and disinfection. These bacteria produced 3.13-fold more biofilm mass compared to the sum of biofilm masses in monoculture. The present study confirms that the observed community synergy results from dynamic social interactions, encompassing commensalism, exploitation, and amensalism. *M. lacticum* appears to be the keystone species as it increased the growth of all other species that led to the synergy in biofilm mass. Interactions among the other three species (in the absence of *M. lacticum*) also contributed towards the synergy in biofilm mass. Biofilm inducing effects of bacterial cell-free-supernatants were observed for some combinations, revealing the nature of the observed synergy, and addition of additional species to dual-species combinations confirmed the presence of higher-order interactions within the biofilm community. Our findings provide understanding of bacterial interactions in biofilms which can be used as an interaction–mediated approach for cultivating, engineering, and designing synthetic bacterial communities.

## Introduction

Biofilms are increasingly recognised as an important concern for multiple industries, affecting food production and safety, water supply, health, industrial processes, and the marine sector where the presence of biofilms causes economic impact of billions of USD per year [1]. Almost all biofilms in natural and industrial settings are composed of multiple and often diverse species that interact with each other and with the environment in a variety of ways as part of the “struggle for existence” [2, 3] and establish several associations ranging from positive interactions (e.g., cooperation +/+ and commensalism +/0 to competition −/−, amensalism 0/−, and exploitation +/− [4–6].

Multispecies biofilms in the dairy industry can contain both pathogens and food spoilers. These biofilms have implications for the safety and quality of food products and economy overall because of their association with enhanced production of spoilage enzymes and toxins [7–9]. Bacteria recovered from food contact surfaces after cleaning and disinfection (C&D) have indeed been shown to interact with each other in a variety of ways that may have implications for persistence and tolerance of these biofilms against disinfectants [10–12].

Despite the awareness of the significance of bacterial interactions in biofilms and their consequences, our current understanding of interspecies interactions – or even the principles governing these interactions in general – is limited, which is mainly because bacterial interactions are complex to study [13]. Outcomes of bacterial pairwise interactions have been used to predict the structure and function of a number of simplified bacterial communities [14, 15]. However, pair-wise interactions do not take into account another type of interaction, termed higher-order interaction, in which the interaction between two species is modulated by one or more other species [16]. Community dynamics are often affected by one or few individual species termed keystone species. A keystone species is a species which, regardless of its frequency, has a significant effect on the ecology, survival and function of other species [17]. Thus, studying variability and strength of both pair-wise and higher-order interactions is imperative to understand drivers of species coexistence in diverse communities [18]. This is not only fundamentally interesting, but this knowledge is important to predict stability and functionality of the community, its evolutionary dynamics and bottom-up biological functions in a range of contexts including controlling biofilms on food contact surfaces.

In our previous work [19], we characterised 140 reproducible four-species biofilm communities on stainless steel (SS) that comprised bacteria previously recovered from the surface of a dairy pasteuriser after routine industrial C&D practices [20]. Out of the 11 four-species combinations that showed synergy (higher biofilm mass in co-culture than the sum of the monoculture biofilm masses) in four-species biofilms, five combinations had three species in common: *Stenotrophomonas rhizophila*, *Bacillus licheniformis*, and *Microbacterium lacticum*. A study on bacterial ecology of biofilms on the surface of a milking machine also reported coexistence of these three species in multispecies biofilms [21]. Strong synergy in biofilm formation was observed in a four-species biofilm community that included these three species and *Calidifontibacter indicus* [19]. However, the re-organisation of bacterial interactions between pairwise cultures and larger communities remained largely unknown. From an ecological perspective, understanding the role of individual species in observed synergy, stability, and community assembly is crucial for a mechanistic understanding of microbial synergistic interactions. Here, we used a bottom-up approach and disentangled the interspecies interactions and growth dynamics in the four-species community by analysing all pairwise and higher order interactions (e.g., three-species interactions and four-species together) between the component species. Overall, this is the first study on dairy isolates which provides an in-depth understanding of the role of various social interactions – from commensalism to exploitation – and assesses the significance of keystone species within a multispecies biofilm community for stability, co-existence and perhaps better survival. Knowledge on these specific bacterial interspecific interactions and the role of keystone species may translate to other bacterial interactions and deepen our understanding of bacterial ecology in general.

## Materials and methods

### Bacterial strains

A total of 4 bacterial strains belonging to different species were used in this study. These strains, previously isolated from the surface of a dairy pasteuriser following routine C&D, were named *S. rhizophila* (B68), *B. licheniformis* (B65), *M. lacticum* (B30), and *Calidifontibacter indicus* (B44) according to Maes et al. [20]. For simplicity, throughout this study, the strains are referred to as *S. rhizophila* (SR), *B. licheniformis* (BL), *M. lacticum* (ML), and *C. indicus* (CI). Hereafter, all species will be represented by the above-mentioned abbreviations in case of combinations and names where single species are to be mentioned. All strains were grown in a general-purpose medium (Brain-Heart-Infusion, BHI) at 30 °C and stored as freezer stocks at −70 °C until use. These 4 species were part of one four-species combination which is hereafter named as follows: SR-BL-ML-CI. This combination of species showed a 3.13-fold increase in biofilm mass. These previous findings are shown in Supplementary Fig. S1.

### Biofilm formation on polystyrene

Biofilms were grown in 96-well polystyrene microtiter plates (Coster 3596, Corning Inc., Corning, NY, USA) using the method described by Oh, CHEN and KANG [22] with some modifications. Bacterial species were incubated overnight (~16 h) under static conditions in 10 mL BHI broth at 30 °C followed by appropriate dilution in fresh BHI to the OD_595_ value of 0.05 for all strains using a Multiskan ™ FC Microplate Photometer (Thermo Fisher Scientific). A total of 160 μL was used as the inoculum volume for single-species biofilms, whereas equal volumes of all species were mixed to a total volume of 160 μL in case of dual- three- and four-species biofilms as previously reported [23]. The microtiter plates were incubated under static conditions at 30 °C for 24 h and biofilms were stained with 0.1% (w/v) crystal violet (CV) and absorbance was measured after solubilising CV with 33% glacial acetic acid at 595 nm (Abs_595_).

### Effects of spent culture supernatants on biofilm formation

Cell-free supernatant (CFS) of each species was produced by filtering the planktonic fractions produced overnight in BHI through a 0.2 μm filter (Whatman, Germany). For each mixed-species biofilm combination, each species was one by one replaced with its CFS to determine the effect of its growth metabolites on the biofilm mass produced by the other species. For supernatant studies, biofilms were grown as described earlier and equal volumes of CFS were used instead of viable cells in BHI for each species in the mixture. The addition of CFS in certain combinations, instead of viable cells suspended in BHI, diluted the growth medium and thus reduced the available nutrients for growth which we termed ‘the dilution effect’. In some combinations where a decrease in biofilm mass was observed due to the presence of CFS of the other species, comparative controls were performed by replacing CFS with the same volume of sterile water in the respective combination to test if the observed effect could be attributed to the dilution effect. CFS was more diluted in higher communities, where the CFS of one species was combined with two to three viable species, compared to dual cultures. This dilution effect could have impeded the full explanatory potential of CFS, highlighting a limitation of this study.

### Biofilm development on SS coupons and cell counting

Bacterial growth dynamics in the four-species biofilm combination (SR-BL-ML-CI) were studied every 4 h over the period of 24 h. Biofilms were grown on SS coupons (AISI 304 grade: 30 × 15 mm dimension) in the presence of BHI and cow’s skim milk (SM) (FrieslandCampina, Belgium). For interactions in two- and three-species combinations, all biofilms on SS were grown for 24 h and only in BHI. Selective counting of each species in all mixed-species biofilm combinations was carried out on species-specific media plates that were developed based on the selection of temperature, type of the growth media, and different concentrations of antibiotics (see Supplementary Material File S1). We confirmed that none of the selective counting regimes affected the count of bacteria, compared to their growth in monoculture on BHI agar plates. In addition to counting individual cells recovered from mixed-species biofilms, cells in the mixed-species planktonic fraction both in BHI and SM were also quantified at six time points (4 h, 8 h, 12 h, 16 h, 20 h and 24 h after coincubation). Biofilms were grown on SS using 6-well microtiter plates (Costar® 3516) by placing the coupons horizontally in each well containing 5 mL BHI or SM followed by incubation at 30 °C until the required incubation period was attained.

All bacterial species were grown overnight and diluted in BHI or SM to the concentration ~ 1 × 10^5^/mL. Bacteria were allowed to form biofilms in single culture as well as cocultures. For single-species cultures, 5 mL were added to 3 wells of a 6-well microtiter plate (Coster 3516, Corning Inc., Corning, NY, USA). For any co-cultured biofilm combinations, all diluted bacterial cultures were pooled to a final total volume of 5 mL. After the required period of incubation, SS coupons were removed from the medium using sterile forceps and immersed in sterile distilled water for three consecutive times to remove any loosely attached cells. The coupons were then transferred to 9 mL sterile saline solution and subjected to the combination of sonication (10 min) and vortexing (2 min) [24] for the efficient removal of cells from the SS coupons. Cells were subsequently counted by serial dilution and culturing on species-specific media plates. The pH values of the planktonic fractions of single species and all combinations were also measured at 24 h.

### Scanning electron microscopy (SEM)

SEM was used to observe bacterial spatial organisation in single and mixed-species biofilms on SS coupons in BHI after 24 h. Briefly, the coupons were first rinsed with sterile double distilled water to remove loosely attached cells and then double fixed with 2.5% glutaraldehyde (Sigma-Aldrich, Saint Louis, Missouri, USA) in 0.1 M sodium cacodylate (Sigma-Aldrich, Saint Louis, Missouri, USA) (pH 7.4) for > 8 h. Post fixation of biofilm on SS was performed in 1% osmium tetroxide in 0.1 M cacodylate for 1-2 h. After fixation, the coupons were washed for three consecutive times (5 min each) with 0.1 M cacodylate. Dehydration was performed in graded alcohol solution (30–100% v/v solutions). Finally, the SS coupons were dehydrated with liquid CO_2_ in a Hitachi Model HCP-2 critical point dryer. Hitachi Model E-1010 ion sputter was used to coat the dehydrated samples with gold-palladium for 4–5 min and biofilms were observed in Zeiss Crossbeam 540 FIB-SEM.

### Statistical analyses

Each experiment was repeated three times on different occasions with three replicates in each trial. In each of the three experiments, the optical density was measured from four wells in each of the three replicate microtiter plates per sample to determine bacterial biofilm mass in both single and mixed cultures. Statistical significance was determined by one-way analysis of variance (ANOVA) followed by a Duncan’s Multiple Range Test using SPSS v.23. Differences between means with *p* < 0.05 were denoted across values of each species with letters. Paired *t*-tests were conducted to determine significant differences between bacterial cell numbers between monocultures and cocultured biofilms using GraphPad Prism 9. Values of *P* ≤ 0.05 were considered statistically significant.

## Results

Strong biofilm synergy was found among 3 bacteria (*S. rhizophila*, *B. licheniformis* and *M. lacticum*) when co-cultured, showing an overall 2.65-fold increase in the biofilm mass, compared to the sum of monoculture biofilm masses. The addition of *C. indicus* as the fourth species further increased the biofilm mass by 21%. We investigated the interactions by studying all pair-wise and higher-order interactions. Based on the observed changes in bacterial cell count in single and all mixed-cultures, bacterial interactions were classified as commensalism (+/0) (an interaction in which cell count (growth) of one species increases without any effect on the growth of the other species), amensalism (0/−) (an interaction in which one species reduces the cell count (growth) of the other species without having any effect on its own growth), and exploitation (+/−) (an interaction in which cell count (growth) of one species increases with a negative effect on the growth of the other species), as defined previously [25]. We did not observe other types of interactions among the species interacting in pairs (e.g., reciprocal positive effects/mutualism (+/+) or altruism (−/0 + ).

### Pairwise interactions in terms of cell count and biofilm mass

**Fig. 1:**
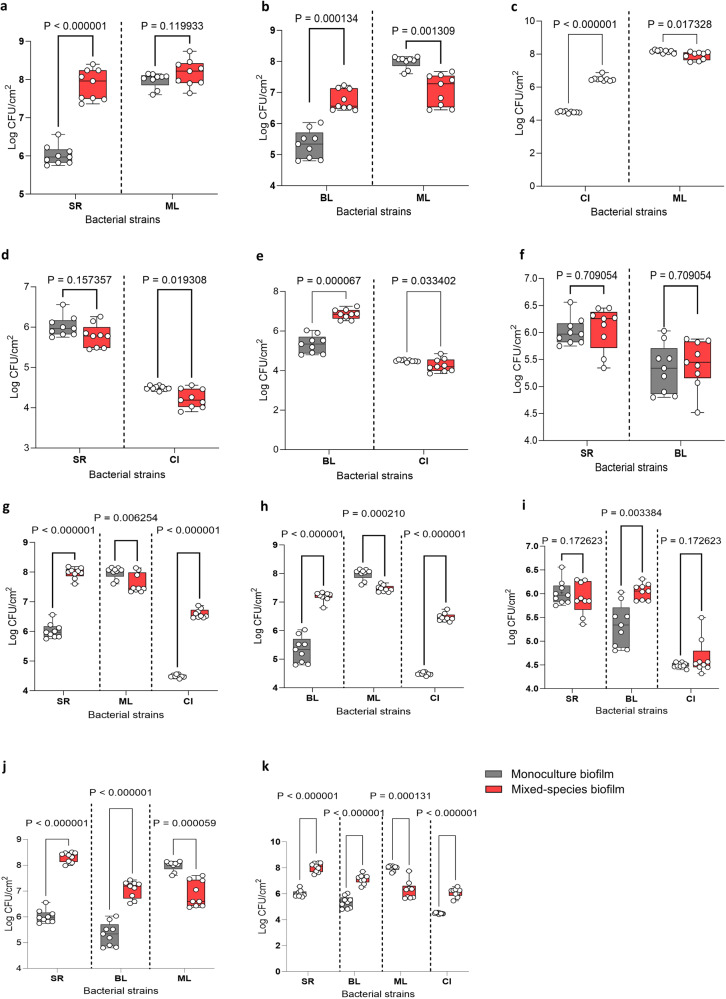
Interspecies interactions in all possible dual-, three- and four-species biofilm combinations are shown for the four species: *Stenotrophomonas rhizophila* (SR), *Bacillus licheniformis* (BL), *Microbacterium lacticum* (ML) and *Calidifontibacter indicus* (CI). The outcome of pairwise (**a**–**f**), three-species (**g**–**j**), and the four-species community (**k**) is shown in terms of changes in individual cell numbers on stainless steel (log CFU/cm^2^) in monoculture and co-culture biofilms after 24 h. Biofilms were grown on stainless surface in brain-heart-infusion medium. Statistical difference in cell counts between single and co-culture biofilms for each strain was determined using a two-tailed paired *t*-test on GraphPad Prism version 9.4.1 and all corresponding two-tailed *P* value are shown where significance was determined at *p* < 0.05.

**Fig. 2:**
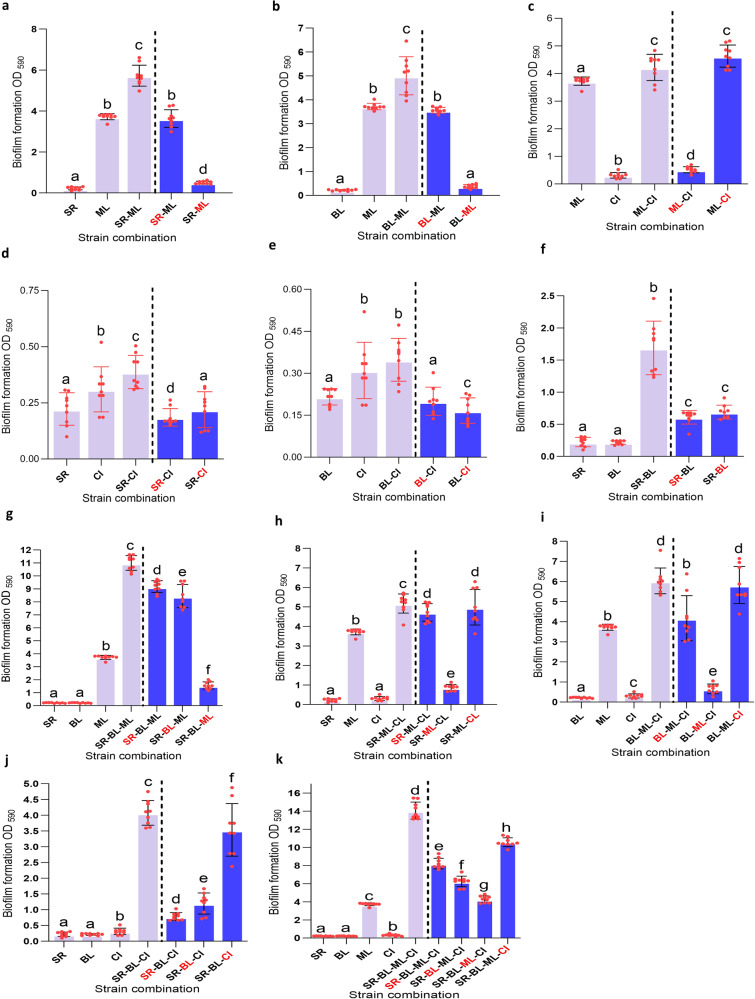
Interspecies interactions in all possible dual-, three- and four-species biofilm combinations are shown for the four species: *Stenotrophomonas rhizophila* (SR), *Bacillus licheniformis* (BL), *Microbacterium lacticum* (ML) and *Calidifontibacter indicus* (CI). The outcome of pairwise (**a**–**f**), three-species (**g**–**j**), and the four-species community (**k**) is shown in terms of changes in biofilm mass (OD_595_) that was quantified using the 96-well microtiter plate method. All biofilms were developed on stainless surface in brain-heart-infusion medium for 24 h. Statistical significance was determined by one-way analysis of variance (ANOVA) followed by a Duncan’s Multiple Range Test using SPSS v.23. Significant differences (*P* < 0.05) between the strains are indicated with different letters (**a**, **b**, **c**, **d**) above the bars.

We first tested all possible pairwise interactions to examine their effect on individual cell numbers and total biofilm mass, as shown in Figs. 1 and 2, respectively. We also assessed whether CFS (derived from bacterial monocultures grown overnight) of one species affected the biofilm mass produced by the remaining species present (Fig. 2). Images of biofilm mass for all combinations stained with CV are shown in Supplementary Fig. S2.

**Fig. 3:**
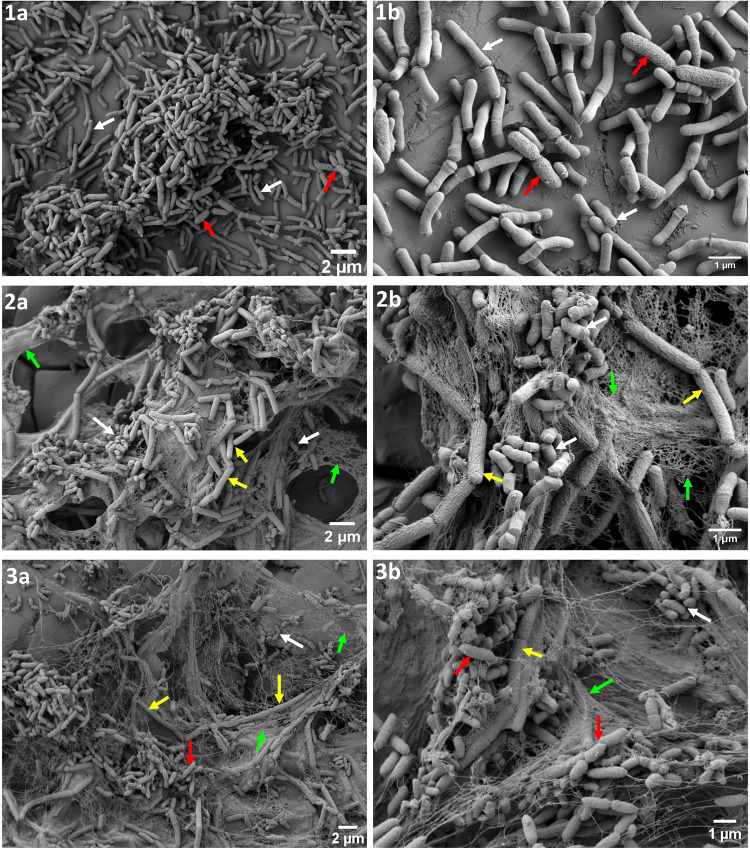
Scanning electron microscopy images of dual- and three-species biofilms formed by *Stenotrophomonas rhizophila*, *Bacillus licheniformis*, and *Microbacterium lacticum* on stainless steel surface in brain-heart-infusion medium after 24 h. **1a**, **b** Biofilm-based association between *S. rhizophila* and *M. lacticum* where both cells are shown by red and white arrows, respectively. The middle part of the Fig. (2**a**, **b**) shows association between *B. licheniformis* and *M. lacticum*, where these species are indicated by yellow and white arrows. Extracellular polymeric substances (EPS) are shown by green arrows. The last part of the Fig. (**3a**, **b**) shows biofilm-based association among three species in the co-culture biofilm where *S. rhizophila*, *B. licheniformis*, and *M. lacticum* are shown by red, yellow and white arrows, respectively. EPS are shown by green arrows.

Viable cells of *M. lacticum* stimulated growth of all other species in pair-wise interactions. *S. rhizophila* established a symbiotic relationship (commensalism) with *M. lacticum* in which its growth was increased by ~ 2 log CFU/cm^2^ (Fig. 1a). Synergy in biofilm biomass (Fig. 2a) also indicated more growth and/or matrix production in the coculture. The SEM image showed that *M. lacticum* formed a layer of cells directly on the steel surface onto which cells of *S. rhizophila* settled (Fig. 3: 1a, b). *M. lacticum* was exploited by *B. licheniformis* (Fig. 1b) and C*. indicus* (Fig. 1c) where the cell count of the two latter species was increased by 1.4 and 2 log CFU/cm^2^. In case of BL-ML, SEM images show high extracellular matrix production by *B. licheniformis* cells (Fig. 3; 2a, b). Interactions between *S. rhizophila* and *C. indicus*, as shown in Fig. 1d, can be characterised as amensalism where *S. rhizophila* significantly reduced, although slightly, the growth of *C. indicus* without any significant change in its own growth. *B. licheniformis* appeared to lead to an exploitative relationship with *C. indicus*, similar to its relationship with *M. lacticum* (Fig. 1e). The interaction between *S. rhizophila* and *B. licheniformis* (combination SR-BL) appeared to be neutral (no fitness effect) in terms of their cell counts in mono- and mixed cultures (Fig. 1f), which is in contrast to the observed increase in biofilm mass (~ 4-fold) (Fig. 2f). Replacement of viable cells with their CFS did not impact biofilm biomass in most pair-wise interactions. However, it is noteworthy that CFS of *S. rhizophila* and *B. licheniformis* caused an increase of 2.7- and 3.1-fold in biofilm mass produced by *B. licheniformis* and *S. rhizophila*, respectively, compared to their monoculture biofilm mass (Fig. 2f). However, the biofilm mass produced by viable cells of the two species in combination SR-BL was at least > 2-fold higher than the one produced by the combination with CFS of one species. The biofilm mass of *M. lacticum* was increased by 24% in the presence of *C. indicus* CFS which may be due to the absence of competitive effect that was observed in the presence of viable *C. indicus* (Fig. 2C).

### Higher-order interactions in terms of cell count and biofilm mass

Higher-order interactions were studied in all possible three-species and the four-species combinations in terms of individual bacterial cell count and biofilm mass, shown in Figs. 1 and 2, respectively. Effect of CFS of one species on the growth of others was also assessed. We first assessed interspecies interactions in the three-species community (SR-BL-ML) and then added *C. indicus* with all pairs (SR-ML, BL-ML, and SR-BL) to investigate higher-order interactions. Table 1 shows a comparison of cell counts and biofilm mass in all dual- and three-species combinations.

**Table 1:** A comparison of biofilm mass (on polystyrene) and cell counts (on stainless steel) between different dual-, three-, and four-species combinations to assess the effect of adding different species on the interaction of other species present.

Combinations	Biofilm mass (OD_590_)	SR Cell count (log CFU/cm^2^)	BL Cell count (log CFU/cm^2^)	ML Cell count (log CFU/cm^2^)	CI Cell count (log CFU/cm^2^)
*Group 1*					
1-2-3	10.99 ± 0.53^a^	8.31 ± 0.18^a^	7.06 ± 0.31^a^	6.88 ± 0.47^a^	-
1-2	1.69 ± 0.39^b^	6.07 ± 0.38^b^	5.42 ± 0.41^b^	-	-
1-3	5.72 ± 0.48^c^	7.86 ± 0.37^c^	-	8.17 ± 0.32^b^	-
2-3	5.0 ± 0.75^d^	-	6.78 ± 0.31^c^	7.08 ± 0.47^a^	-
*Group 2*					
1-3-4	5.18 ± 0.47^a^	7.96 ± 0.17^a^	-	7.61 ± 0.30^a^	6.59 ± 0.14^a^
1-3	5.72 ± 0.48^b^	7.86 ± 0.37^a^	-	8.17 ± 0.32^b^	-
1-4	0.39 ± 0.06^c^	5.78 ± 0.27^b^	-	-	4.22 ± 0.22^b^
3-4	4.22 ± 0.44^d^	-	-	7.89 ± 0.23^b^	6.49 ± 0.14^a^
*Group 3*					
2-3-4	6.0 ± 0.60^a^	-	7.19 ± 0.15^a^	7.46 ± 0.11^a^	6.46 ± 0.13^a^
2-3	5.0 ± 0.75^b^	-	6.78 ± 0.31^b^	7.08 ± 0.47^b^	-
2-4	0.35 ± 0.07^c^	-	6.85 ± 0.23^b^	-	4.26 ± 0.31^b^
3-4	4.22 ± 0.44^d^	-	-	7.89 ± 0.23^c^	6.49 ± 0.14^a^
*Group 4*					
1-2-4	4.0 ± 0.37^a^	5.89 ± 0.31^a^	6.04 ± 0.16^a^	-	4.65 ± 0.35^a^
1-2	1.69 ± 0.39^b^	6.07 ± 0.38^b^	5.42 ± 0.41^b^	-	-
1-4	0.39 ± 0.06^c^	5.78 ± 0.27^a^	-	-	4.22 ± 0.22^b^
2-4	0.35 ± 0.07^c^	-	6.85 ± 0.23^c^	-	4.26 ± 0.31^b^
*Group 5*					
1-2-3-4	14.06 ± 0.88^a^	8.03 ± 0.33^a^	7.28 ± 0.33^a^	6.20 ± 0.52^a^	6.09 ± 0.35^a^
1-2-3	10.99 ± 0.53^b^	8.31 ± 0.18^b^	7.06 ± 0.31^a^	6.88 ± 0.47^b^	-
1-3-4	5.18 ± 0.47^c^	7.96 ± 0.17^c^	-	7.61 ± 0.30^c^	6.59 ± 0.14^b^
1-2-4	4.0 ± 0.37^d^	5.89 ± 0.31^d^	6.04 ± 0.16^b^	-	4.65 ± 0.35^c^
2-3-4	6.0 ± 0.60^e^	-	7.19 ± 0.15^a^	7.46 ± 0.11^c^	6.46 ± 0.13^b^

Statistical significance was determined by one-way analysis of variance (ANOVA) followed by a Duncan’s Multiple Range Test using SPSS v.23. Values with different letters indicate statistically significant (*P* ≤ 0.05) differences between values within a single group. SR *Stenotrophomonas rhizophila*, BL *Bacillus licheniformis*, ML *Microbacterium lacticum*, CI *Calidifontibacter indicus*.

**Fig. 4:**
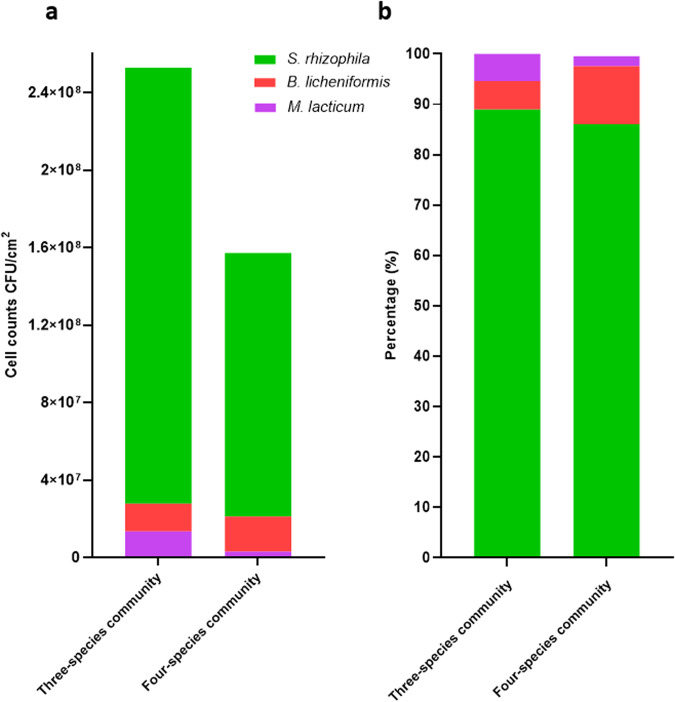
Absolute and relative abundances of the three bacterial strains. The three bacterial strains (*Stenotrophomonas rhizophila*, *Bacillus licheniformis*, and *Microbacterium lacticum*) in the three- and four-species biofilm communities are shown in **a** and **b**, respectively). Absolute and relative abundances of three species was determined with the addition of *C. indicus*. The biofilms were developed on the surface of stainless steel in BHI for 24 h.

There was an overall 2.65-fold increase in the biofilm mass of the combined biofilm community SR-BL-ML, compared to the sum of monoculture biofilm masses. In SR-BL-ML, *S. rhizophila* showed the highest absolute abundance (8.31 log CFU/cm^2^) followed by *B. licheniformis* (7.0 log CFU/cm^2^) and *M. lacticum* (6.9 log CFU/cm^2^) (Table 1 and Fig. 4a). SEM images also confirmed the abundance of *S. rhizophila* and matrix production by *B. licheniformis* as shown in Fig. 3a, b. CFS of *B. licheniformis* and *S. rhizophila* significantly increased the biofilm mass of combination SR-ML and BL-ML, respectively (Fig. 2g). The effect of the supernatants from these two species on each other was in line with their pair-wise interaction (SR-BL). Growth of *S. rhizophila* increased from 7.86 in SR-ML to 8.31 log CFU/cm^2^ in SR-ML-BL probably due to *B. licheniformis* in the presence of *M. lacticum* because *B. licheniformis* had no influence on the growth of *S. rhizophila* in SR-BL. This indicated that pairwise outcomes are further affected by the addition of a third species. The addition of *C. indicus* affected the relative and absolute abundance of the other three species compared to the three-species biofilm (Fig. 4b). Interestingly, CFS of *C. indicus* significantly increased the biofilm mass of combination BL-ML and SR-BL (Fig. 2 i, j).

Cell counts of all species in the four-species biofilm compared to their monoculture biofilms is shown in Fig. 1k. Biofilm mass of the community containing three viable species with CFS of the fourth species was compared with biofilm mass of the three-species community (Fig. 2k; Table 1). When CFS of *S. rhizophila* was present in combination BL-ML-CI, biofilm mass was increased by 36.3% compared to the biofilm of the three-species combination without the CFS of *S. rhizophila*. Similarly, when CFS of *B. licheniformis* was used in combination SR-ML-CI replacing its live cells, biofilm mass increased by 21% compared to the biofilm mass formed by the biofilm community SR-ML-CI. Biofilm mass of combination SR-BL-CI was increased by only 5.1% in the presence of the CFS of *M. lacticum*. These results further confirmed that the presence of *M. lacticum* in viable form (as opposed to its CFS) played a key role in the observed synergy, which resulted in a 71% increase in the biofilm mass of the SR-BL-CI combination. Notably, *M. lacticum* was involved in most synergistic pairs and trios, such as SR-ML, BL-ML, SR-BL-ML, and BL-ML-CI. Thus, *M. lacticum* played the role of keystone species in the community by stimulating the growth of all other species. In terms of numbers of bacterial cells, all three species achieved fitness advantages, as assessed by induced growth, as part of the four-species biofilm, except *M. lacticum*, of which growth was reduced due to the exploitative relationship with *B. licheniformis* and *C. indicus*. Overall, synergy in biofilm mass in the four-species biofilm was not only related to pairwise interactions, but also through interactions in which one species modulated the interactions between two pairs of species.

### pH of the planktonic fractions of all combinations

Bacterial interactions are often mediated through environmental modifications (e.g., pH changes) as a result of different metabolic activities which affect the growth of both themselves and also other microbes [26]. The pH measurements of the liquid/planktonic fractions from various  mixed-species biofilm combinations were compared with those of monoculture planktonic fractions after 24 h. (Table 2). Changes in pH seemed to indicate relative proportions of each species. *M. lacticum* was the only species that lowered the pH of BHI to 6, while the growth of both *S. rhizophila* and *B. licheniformis* increased the pH to above 8. pH measurements of the SR-ML and BL-ML combinations (>7.6) and SR-BL-ML (>8) indicate relatively higher growth of *S. rhizophila* and *B. licheniformis* compared to *M. lacticum*. This observation aligns with the relative proportions of each species in these combinations.

**Table 2:** The pH values of the planktonic fraction of pure and different combinations of strains after 24 h.

Strains and combinations	pH of the planktonic fraction ± SD
SR	8.26 ± 0.06
BL	8.04 ± 0.05
ML	6.00 ± 0.03
CI	7.46 ± 0.15
SR - ML	7.62 ± 0.13
BL - ML	7.70 ± 0.18
SR - BL	8.08 ± 0.09
SR - BL - ML	8.18 ± 0.10
SR - CI	8.16 ± 0.03
BL - CI	7.85 ± 0.10
ML - CI	6.46 ± 0.15
SR - ML - CI	7.45 ± 0.09
BL - ML - CI	7.63 ± 0.07
SR - BL - ML - CI	8.01 ± 0.11

SR *Stenotrophomonas rhizophila*, BL *Bacillus licheniformis*, ML *Microbacterium lacticum*, CI *Calidifontibacter indicus*. The values represent mean values ± standard deviation obtained in three independent experiments.

### Species growth dynamics in the four-species community

**Fig. 5:**
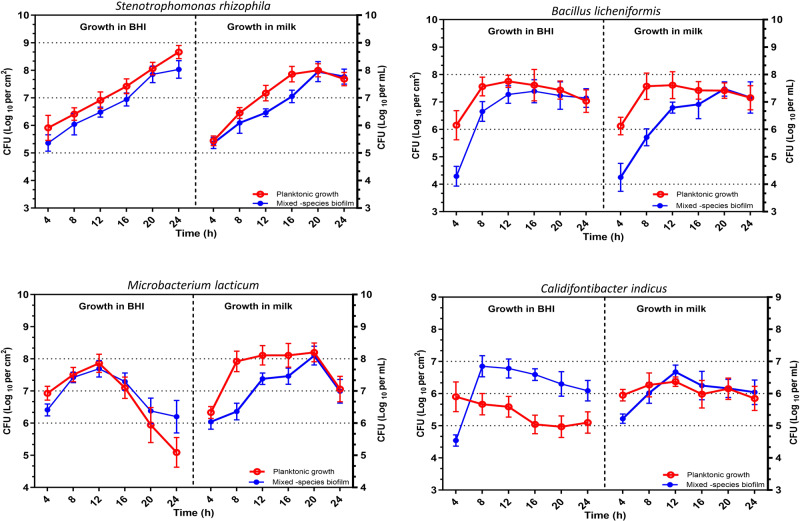
Cell numbers of biofilm- (blue) and planktonic-associated (red) fractions of each species in a model four-species biofilm community on stainless steel coupons (AISI 304 grade) in skim milk and brain-heart-infusion medium. The estimated cell numbers/cm^2^ in biofilms and per mL in planktonic fraction of each strain in the four-species biofilm and the associated planktonic fractions comprising *Stenotrophomonas rhizophila*, *Bacillus licheniformis*, *Microbacterium lacticum*, and *Calidifontibacter indicus* were determined at six time points (4 h, 8 h, 12 h, 16 h, 20 h and 24 h after coincubation) based on selective media plates. Each point represents the mean of three replicates, and the vertical lines indicate the standard deviation.

**Fig. 6:**
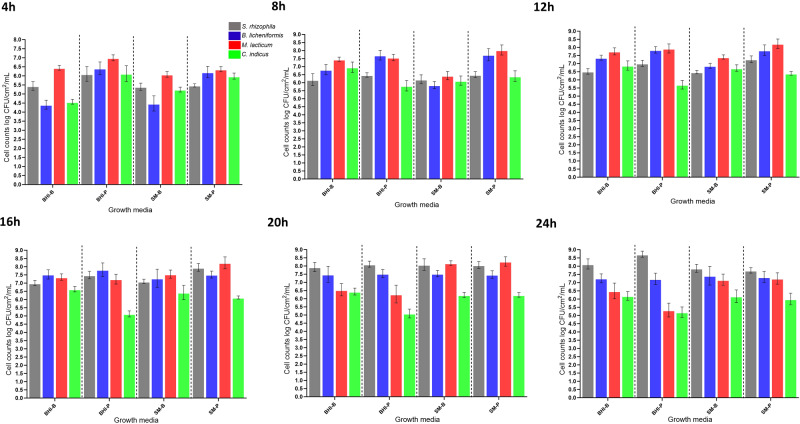
A comparison of the cell numbers of the four bacterial species (*Stenotrophomonas rhizophila*, *Bacillus licheniformis*, *Microbacterium lacticum*, and *Calidifontibacter indicus*) in biofilm (CFU/cm^2^) and planktonic fraction (CFU/mL) on stainless steel coupons (AISI 304 grade) in brain-heart-infusion (BHI) and skim milk (SM) at six time points (4 h, 8 h, 12 h, 16 h, 20 h and 24 h after coincubation) based on selective media plates. Each point represents the mean value of cell counts from three biological replicates. Error bars represent SD of the mean. BHI-B and BHI-P indicate cell counts in biofilm and planktonic fractions in brain heart infusion, respectively; whereas, SM-B and SM-P indicate cell counts in biofilm and planktonic fractions in skim milk, respectively. Grey, blue, red, and green bars indicate *S. rhizophila*, *B. licheniformis*, *M. lacticum*, and *C. indicus*, respectively.

Bacterial growth dynamics in terms of bacterial cell counts were determined for each species in the four-species biofilm on SS and the associated planktonic fractions in BHI and SM over the period of 24 h (Figs. 5 and 6). Relative proportions of each species (%) in the four-species biofilm community on SS in the presence of BHI and SM is shown in supplementary Fig. S3. In general, for biofilms in BHI, a marked increase in cell numbers was observed for *S. rhizophila* and *B. licheniformis*, until 20 and 16 h, respectively, whereas *C. indicus* and *M. lacticum* decreased after 8 and 12 h, respectively. For *S. rhizophila*, *B. licheniformis*, and *M. lacticum*, higher cell numbers were observed in the BHI-based planktonic fraction compared to the biofilm fraction up to 24, 20, and 12 h, respectively. However, the differences were not statistically significant at all time points. From 8–24 h, the cell numbers of *C. indicus* in the mixed-species biofilm were significantly higher than in the mixed-species planktonic fraction in BHI. The species proportion pie charts show that with an increase in *S. rhizophila* and *B. licheniformis* in the mixed species biofilm there was a constant decrease in the proportion of *M. lacticum* (Fig. S3). *M. lacticum* grew very fast from 0 to 4 h and became the most dominant community member (89% abundance), followed by *S. rhizophila* (8.9%). After 24 h, *M. lacticum* was proportionally 2% of the whole biofilm community compared to *S. rhizophila* which became dominant with 85.7% cells in the community followed by *B. licheniformis* (11.4% cells). Marked differences were observed in the growth and stability of *M. lacticum* in the four-species biofilms when comparing with in BHI and SM as *M. lacticum* cell numbers increased or remained constant in the biofilm community in SM until 20 h (1.5 × 10^8^ cells/cm^2^) compared to its growth in BHI where it started decreasing after 12 h from 3.5 × 10^6^ cells per/cm^2^. A 24 h biofilm in either SM or BHI was characterised by high relative abundance of *S. rhizophila* with 64.4 and 85.6% of the cells, respectively. However, the stable growth of *M. lacticum* in SM kept *B. licheniformis* at lower proportions from 8–20 h compared to its presence in BHI (Fig. 6). This may be linked to some stability of *M. lacticum* and consequences thereof on the exploitative relationship.

## Discussion

In mixed biofilm communities, bacterial species often gain fitness advantages, evident through enhanced growth and increased biofilm mass when co-existing with other species. Several factors contribute to this advantage. One of the factors is the facilitated exchange of nutrients and waste products within these mixed communities, which can provide distinct growth benefits to specific species [27]. Additionally, interactions between species can modulate the biofilm’s architecture, thereby improving nutrient availability [28]. Our findings further substantiate the presence of dynamic social interactions among species within a single biofilm community. These interactions can have varied implications for the growth and matrix production of the resident species.

SS coupons were used in our trial to simulate the dairy pasteurizer environment from which the species were originally isolated. The four-species combination was grown in both BHI and SM. The growth dynamics in SM are more relevant for the dairy industry, and using different media highlights the consistency of our findings. SM was not used in all trials (e.g., pairwise combinations and trios) due to milk protein coagulation preventing biofilm mass measurement, allowing only for cell counts. Hence, SM and BHI were used exclusively for cell population dynamics in the four-species combination, while BHI was used to determine cell counts and biofilm mass for other combinations.

The results from all possible pairwise interactions among the four species reaffirmed the concept of keystone species in communities. A disproportionately large effect of *M. lacticum* on growth of all three species and on overall synergy in biofilm mass formed by the four-species community strongly indicated the role of *M. lacticum* as a keystone species. Keystone species in a biofilm community–regardless of their proportion – often serve as a trigger of biofilm formation in other species as well as a metabolic facilitator or protector [29–31]. The significance of keystone species in promoting multispecies biofilms [31] and conferring anti-microbial tolerance to the community members [32] has been reported. Exclusion of a single strain – Actinobacteria (*Rhodococcus* or *Microbacterium*) - from a 62-strain community was shown to significantly affect the community diversity and structure [33]. Some keystone species (e.g., *Enterococcus faecalis, Porphyromonas loveana* and *Dialister pneumosintes*) are important drivers of bacterial community composition and their absence affects the abundance of several other bacterial species due to their role in stimulating the growth of other bacteria [34, 35]. In our combination, it is a possibility that *M. lacticum* is the only species which efficiently adheres and forms biofilm on SS and other species interact with *M. lacticum* by adhering to its surface and *B. licheniformis* plays an important role in building up the matrix.

When *M. lacticum* was replaced with its CFS in the SR-ML and BL-ML combinations, there was no observed change in the biofilm mass of *S. rhizophila* and *B. licheniformis*. In a study examining biofilms formed by bacteria isolated from soil, no noticeable effect of the CFS from one bacterium on another was observed in various combinations [23]. One explanation is that the CFS of *M. lacticum* used in this study was derived from overnight growth of monoculture *M. lacticum*, whereas in our model biofilm, *M. lacticum* impacted the growth of *S. rhizophila* in co-culture conditions in a structured biofilm environment. Certain metabolites that mediate interaction can moreover be unstable and degraded in CFS before administration and thus the dynamics of exposure of partner species to metabolites of the other species, produced in monoculture vs co-culture, could be different. A significant increase in the biofilm mass of *B. licheniformis* in the presence of *S. rhizophila* without any significant change in its cell count may be related to stress response. EPS is often produced as a stress-response strategy in bacterial biofilms, and it is exploited by non-EPS producing strains to get protection or to fulfil certain nutritional needs [36, 37].

Here, we also provide evidence of higher-order interactions in the biofilm community. CFS of *S. rhizophila* and *B. licheniformis* possibly contained metabolites which induced cell growth or biofilm formation of either species in the presence of *M. lacticum*. Another example of higher-order interaction was the effect of *C. indicus* on two dual-species combinations: SR-BL and BL-ML. Keeping in mind a pronounced interaction leading to a greater increase in biofilm mass in the dual-species combination SR-BL, it could be inferred that the effect of CFS from *B. licheniformis* and *S. rhizophila* on SR-ML and BL-ML, respectively, was related to the interaction between the two species (*S. rhizophila* and *B. licheniformis*) which was further enhanced in the presence of *M. lacticum*. Another possibility is the generation of new niches by bacteria through secretion of molecules that possibly alter the pH of the microenvironment and thereby affect the growth of both themselves and also other microbes [38]. According to ‘coexistence theory’ which is based on pH sensitivity of bacterial communities, when a certain type of bacteria dominate, the pH is biased to the optimum value of the dominant bacteria, which strongly suppresses the growth of other type of bacteria [39]. In our trial, CFS fractions of *B. licheniformis* or *S. rhizophila* (pH > 8) might have altered the pH, creating an alkaline environment, which might have favoured the growth of either of these species suppressing *M. lacticum*.

**Fig. 7:**
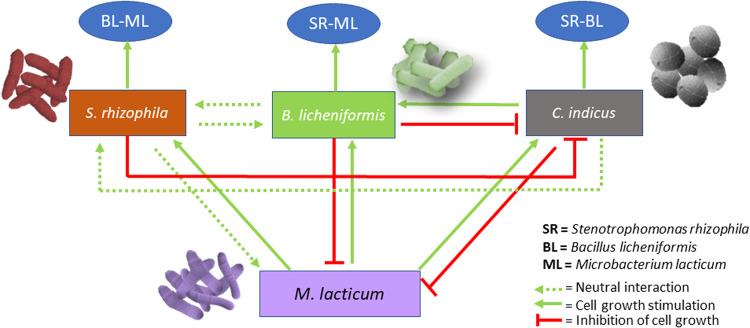
A schematic presentation of bacterial interspecies interactions in a four-species biofilm community on stainless steel in brain-heart-infusion medium comprising *Stenotrophomonas rhizophila* (SR), *Bacillus licheniformis* (BL), *Microbacterium lacticum* (ML), and *Calidifontibacter indicus* (CI). *M. lacticum* is shown to induce growth in all other species, whereas its own growth is negatively affected by *B. licheniformis* and *C. indicus*. *S. rhizophila* is shown to have a neutral interaction with *B. licheniformis*. *C. indicus* is shown to be exploited by *B. licheniformis* as it induces growth of *B. licheniformis* with a negative effect on its own growth. *S. rhizophila*, *B. licheniformis*, and *C. indicus* can be seen to mediate interactions in BL-ML, SR-ML and SR-BL, respectively.

Bacterial community interaction networks are central for understanding the structure and function of microbial communities in natural and industrial settings. It has been a matter of debate over the last one decade whether pairwise interactions are good determinants of community assembly [40] or higher-order interactions should be taken into account to explain community structure [41, 42]. We observed that the outcomes of pair-wise interactions did not provide insights into specific interactions in which a pair-wise interaction was mediated by a third species (e.g., SR-BL-CI). We confirmed the presence of interspecies interaction network among the four species which probably equilibrated all negative pair-wise interactions into a ‘competitive balance’. Nevertheless, there is no evidence that strong higher-order positive effects emerge when specific species pairs, interacting negatively, also interact with other species, which is in line with a previous finding [43]. A study on Zebrafish gut bacterial communities [44] and on bacteria isolated from *Caenorhabditis* elegans intestines [45] showed that bacterial competitive interactions in pairs could not be used to predict species abundances in more complex communities because higher-order interactions dampen pairwise competition. Overall synergy of the four-species community was mainly a result of the growth-promoting effect of *M. lacticum* on other species which further mediated pair-wise interactions. *C. indicus* stimulated growth of *B. licheniformis* which mediated interaction between *S. rhizophila* and *M. lacticum*. The interaction network between the four-species based on our findings is shown in Fig. 7.

Bacterial ratios in SM- and BHI-based four-species biofilm communities did not vary much except the behaviour of *M. lacticum*, which achieved higher cell numbers and remained stable in SM for a longer period of time. Milk-adapted strains of *M. lacticum* have been reported to grow to higher cellular densities in milk compared to other media [46]. Bacterial interspecies interactions depend on the environment and the availability of nutrients. The relative abundance of a bacterial strain at the beginning of co-cultured incubation is not predictive of its colonisation success at later stages [33]. *M. lacticum* was exploited in the community and other species strongly depended on it and gained growth advantages. It is important to highlight that in the mixed-species biofilm on SS in BHI, *M. lacticum* was the predominant species until 12 h. However, after this point, its growth significantly decreased while that of *S. rhizophila* and *B. licheniformis* notably increased. This suggests that the advantages the latter species experienced might have been influenced by *M. lacticum*’s growth dynamics. Later on other species outcompeted *M. lacticum* and dominated the community. Looking at the pH value of the planktonic fractions of the four-species community (pH = 8) and *M. lacticum* monoculture (pH = 6), it is possible that high pH of the medium caused by the growth of *B. licheniformis* and *S. rhizophila* affected the growth of *M. lacticum*.

Further studies at the transcriptional level are required to understand the gene expression patterns of each species in single and different combinations to gain a better understanding of metabolic interactions or any other associations among these community members. However, it appears that targeting *M. lacticum* will disintegrate the observed synergy and this is the point where strategies targeting the whole communities can be developed once the mechanistic basis of these interspecies interactions are understood.

## Conclusion

In our study, the presence of the keystone species in viable form in close association with other cells was mandatory for the observed synergy. Community-level dynamics did not only arise from pair-wise interactions, but also from the influence of other species on many interacting pairs. Thus higher-order ecological effects beyond pairwise interactions may be key to understand interspecific interactions in simple microbial model communities.

Establishing a deeper understanding of bacterial interaction will allow us to better predict the behaviour of bacteria, and to control and manipulate bacterial biofilms for environmental, industrial and clinical purposes. Fluctuating environmental conditions including nutritional status alter the dependency between the two bacterial strains and thus the results obtained in this study should be interpreted with caution.

## Disclaimer

The results and conclusions in this article reflect only the authors’ view. The funding Research Executive Agency (REA), delegated by the European Commission, is not responsible for any use that may be made of the information it contains.

### Supplementary information

Figure S1

Figure S2

Figure S3

Supplementary figure and table legends

Supplementary Material File S1

## Data Availability

The data supporting the results reported in the paper can be accessed through the following link: 10.5281/zenodo.10001928.
