# Supplementary figures and images for: Dynamic social interactions and keystone species shape the diversity and stability of mixed-species biofilms – an example from dairy isolates
Source: ISME Commun. 2023 Nov 15;3:118. doi: 10.1038/s43705-023-00328-3 (PMC10651889; doi:10.1038/s43705-023-00328-3)

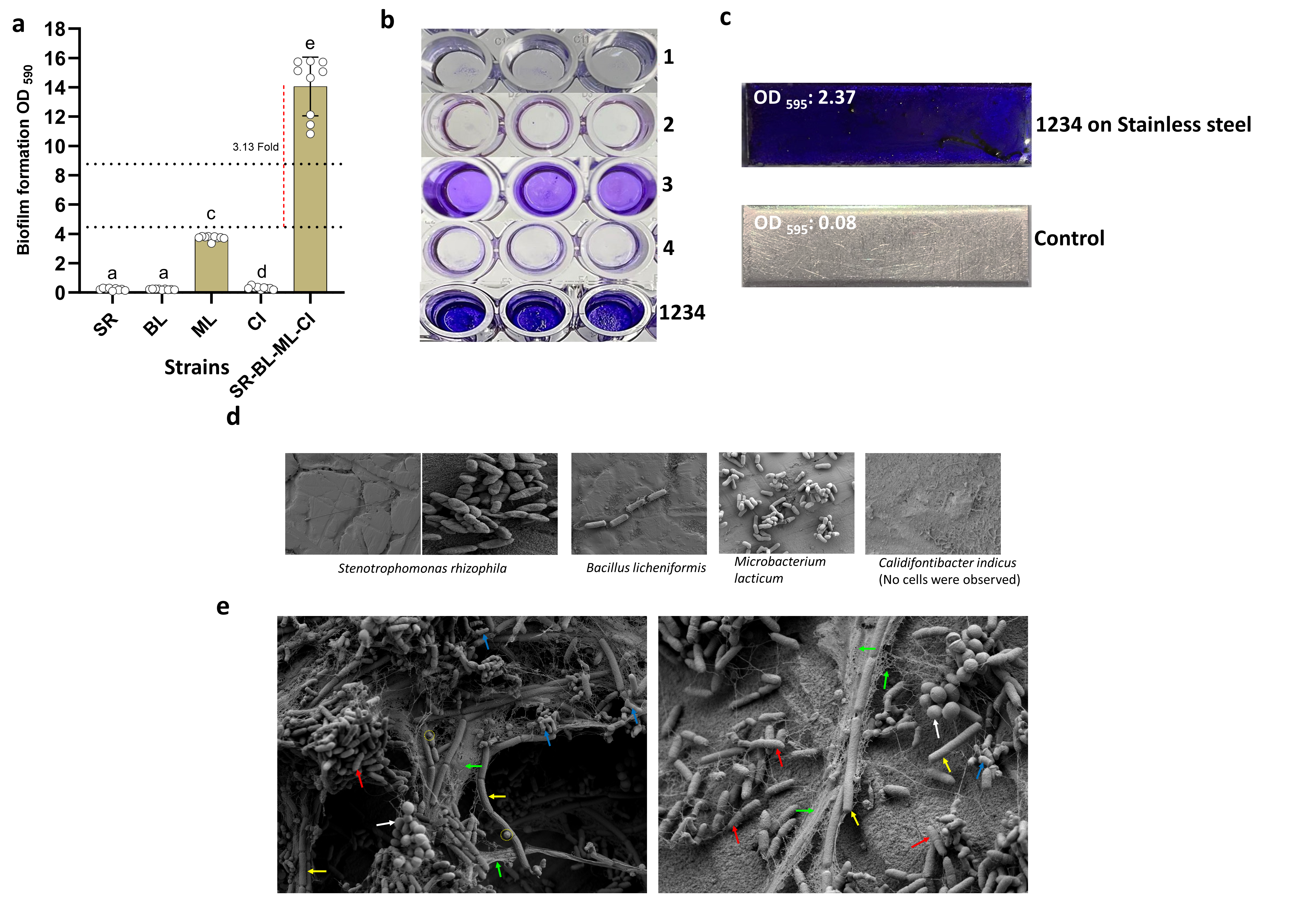

Supplement: Supplementary file 1 — Figure S1 [file 43705_2023_328_MOESM1_ESM.tif]

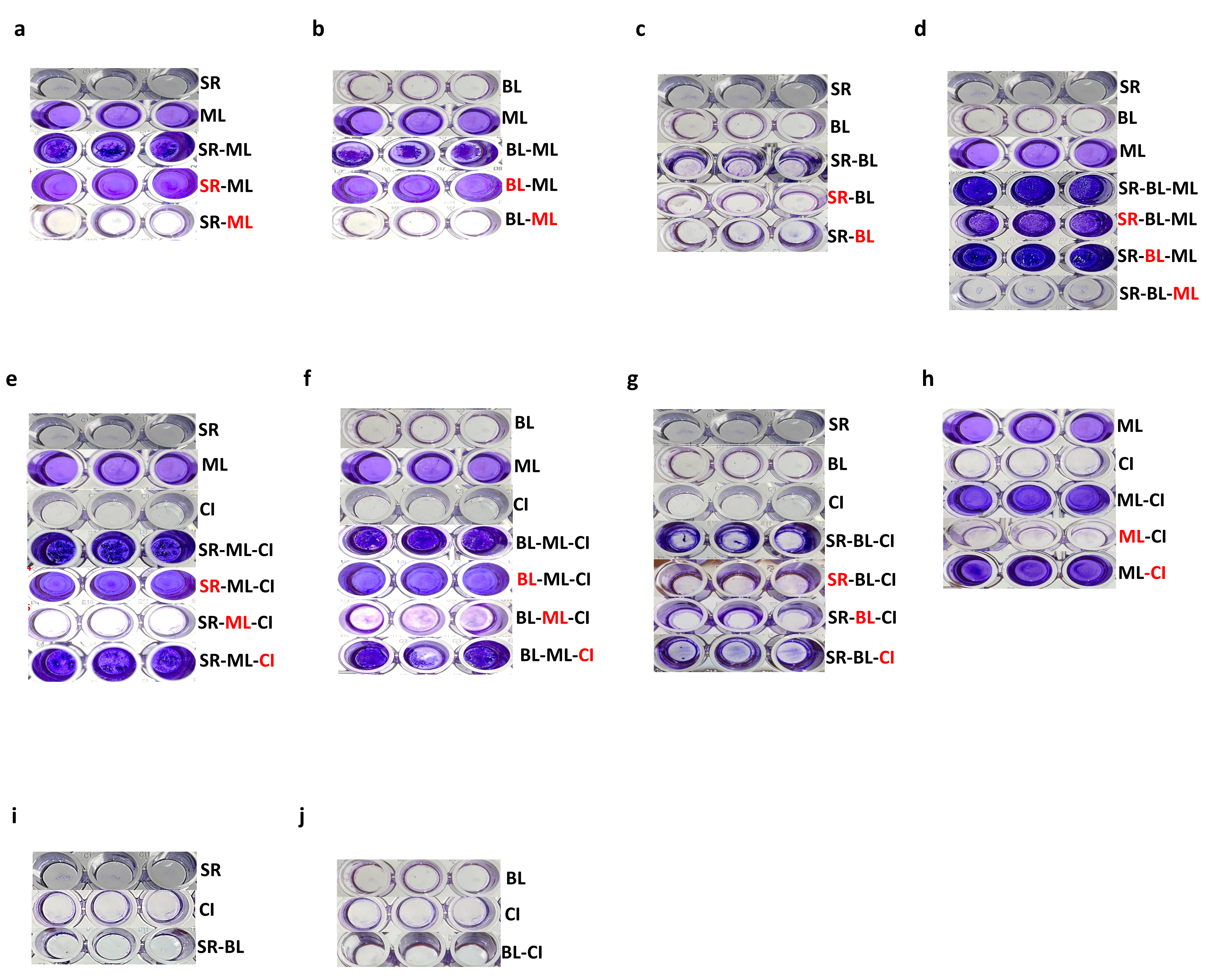

Supplement: Supplementary file 2 — Figure S2 [file 43705_2023_328_MOESM2_ESM.png]

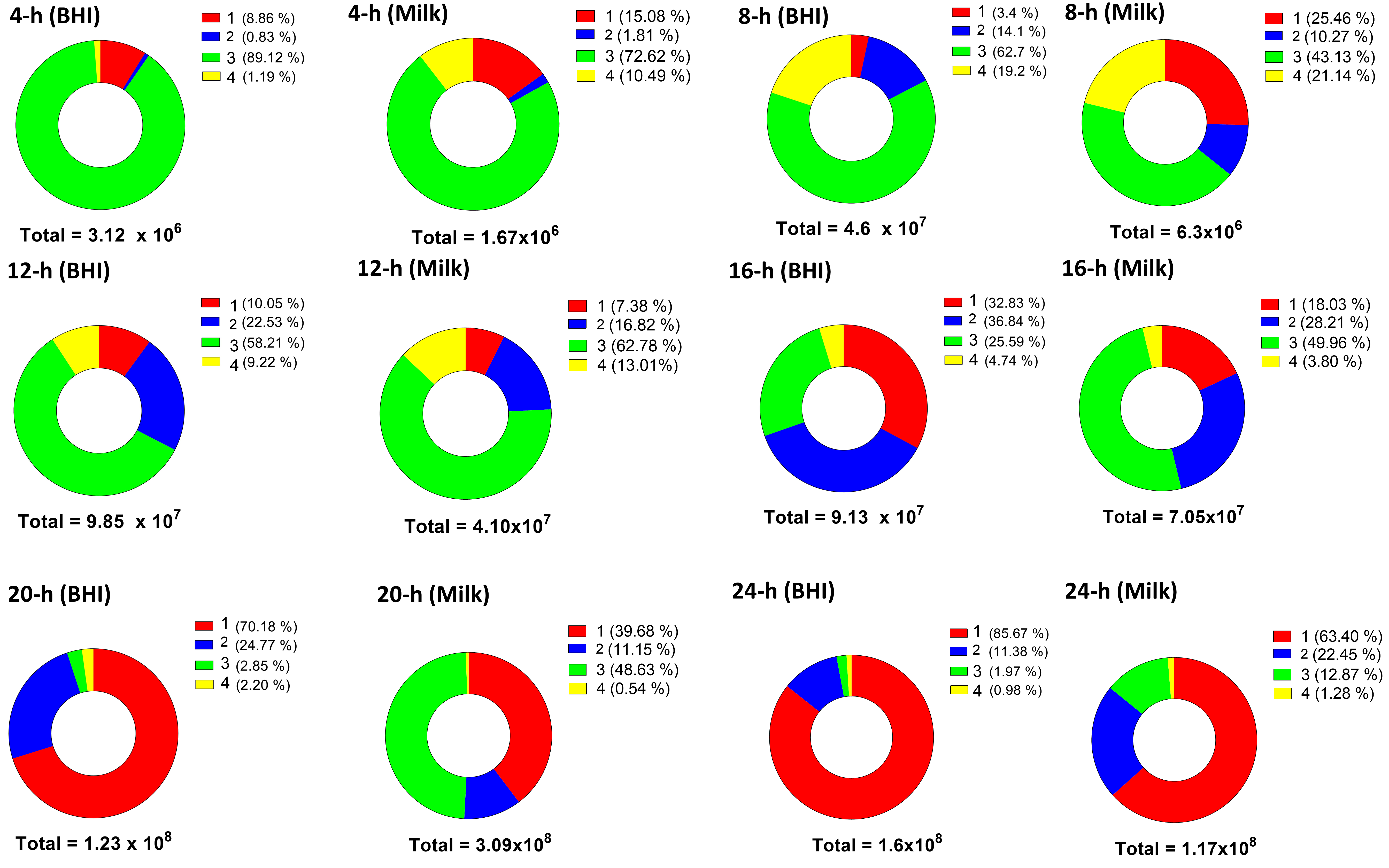

Supplement: Supplementary file 3 — Figure S3 [file 43705_2023_328_MOESM3_ESM.tif]
