# Supplementary material for: Dynamic social interactions and keystone species shape the diversity and stability of mixed-species biofilms – an example from dairy isolates
Source: ISME Commun. 2023 Nov 15;3:118. doi: 10.1038/s43705-023-00328-3 (PMC10651889; doi:10.1038/s43705-023-00328-3)
Supplement: Supplementary file 4 — Supplementary figure and table legends [file 43705_2023_328_MOESM4_ESM.docx]

**Captions of the supplementary figures:**

**Figure S1**. Part a shows synergistic interactions in terms of biofilm mass between *Stenotrophomonas rhizophila* (SR), *Bacillus licheniformis* (BL), *Microbacterium lacticum* (ML), and *Calidifontibacter indicus* (CI) in a four-species biofilm on stainless steel coupons (AISI 304 grade) in brain-heart-infusion broth. Statistical significance was determined by one-way analysis of variance (ANOVA) followed by a Duncan's Multiple Range Test using SPSS v.23. Parts b and c show images of the crystal violet-stained biofilm matrix and cells on the surface of 96-well microtiter plates and stainless steel coupons. The optical density (OD_595_) value of the crystal violet-stained biofilm matrix and cells on stainless coupons is also indicated. Part d shows images of monoculture biofilms for each of the four strains using scanning electron microscopy (SEM). For *S. rhizophila* only a few damaged cell could be seen on the surface, whereas for *C. indicus* no cells could be seen in monoculture. Part e shows mixed-species biofilm of the four species where cells of *S. rhizophila, B. licheniformis, M. lacticum* and *C. indicus* are indicated by red, yellow, blue and white arrows, respectively. *B. licheniformis* spores are also highlighted by a yellow circle. Parts of this images are reproduced from our previous publication (Sadiq et al., 2023).

**Figure S2.** Crystal violet stained wells and biofilm mass photographed for visual representation of biofilm formation by *Stenotrophomonas rhizophila* (SR), *Bacillus licheniformis* (BL), *Microbacterium lacticum* (ML), and *Calidifontibacter indicus* (CI) in monoculture and different mixed-culture biofilm combinations. The influence of replacing each strain one-by-one by its cell-free-supernatant (CFS) in different mixed biofilm combinations is also shown. Strain’s abbreviation written in red indicate presence of CFS of the strain rather than its presence in viable form

**Figure S3**. A comparison of the species proportion in a four-species biofilm on stainless coupons (AISI 304 grade) in brain-heart-infusion broth and skim milk at six time points (4 h, 8 h, 12 h, 16 h, 20 h and 24 h after coincubation). The biological constituents of the four-species biofilm included *Stenotrophomonas rhizophila* (SR), *Bacillus licheniformis* (BL), *Microbacterium lacticum* (ML), and *Calidifontibacter indicus* (CI).

**Supplementary Material File S1**

1. Species-specific growth media plates for selective counting

1.2 Antibiotic concentration in growth media (Brain-heart-infusion medium)
