# Supplementary material for: Dynamic social interactions and keystone species shape the diversity and stability of mixed-species biofilms – an example from dairy isolates
Source: ISME Commun. 2023 Nov 15;3:118. doi: 10.1038/s43705-023-00328-3 (PMC10651889; doi:10.1038/s43705-023-00328-3)
Supplement: Supplementary file 5 — Supplementary Material File S1 [file 43705_2023_328_MOESM5_ESM.docx]

**Supplementary Material File S1**

1. **Species-specific growth media plates for selective counting**

**1.1 Antibiotic concentration**

| **Name of the antibiotic** | **Stock concentration** |
| --- | --- |
| Ciprofloxacine | 0.0004 g or 0.4 mg / mL into 1 M NaOH |
| Polymyxin B sulfate | 1 mg/ mL water |
| Colistine | 1.5 mg / mL water |
| Kanamycin A | 2.5 mg / mL water |

**1.2 Antibiotic concentration in growth media (Brain-heart-infusion medium)**

| **Name of the species** | **Ciprofloxacine** | **Polymyxin B sulfate** | **Colistine** | **Kanamycin A** |
| --- | --- | --- | --- | --- |
| *Microbacterium lacticum* | 13 µl/100 mL media | 110 µl/100 mL media | 33 µl/100 mL media | 40 µl/100 mL media |
| *Calidifontibacter indicus* | 22 µl/100 mL media | 110 µl/100 mL media | **-** | **-** |

***Stenotrophomonas rhizophila* on MacConkey agar No.3 (ThermoFisher Scientific) without antibiotics**

***Bacillus licheniformis* on nutrient agar (ThermoFisher Scientific) without any antibiotic**
